# Clinical, Immunological, Radiographic, and Pathologic Improvements in a Patient with Long-Standing Crohn’s Disease After Receiving Stem Cell Educator Therapy

**DOI:** 10.3390/ijms26157292

**Published:** 2025-07-28

**Authors:** Richard Fox, Boris Veysman, Kristine Antolijao, Noelle Mendoza, Ruby Anne Lorenzo, Honglan Wang, Zhi Hua Huang, Yelu Zhao, Yewen Zhao, Terri Tibbot, Darinka Povrzenic, Mary Lauren Bayawa, Sophia Kung, Bassam Saffouri, Yong Zhao

**Affiliations:** 1 Throne Biotechnologies, Paramus, NJ 07652, USA; rfoxmd@protonmail.com (R.F.);; 2 Fresenius Medical Care North America, Waltham, MA 02451, USA; 3 Life Line Stem Cell Tissue, Cord Blood Bank, New Haven, IN 46774, USA; 4 Valley Radiology Imaging, Los Gatos, CA 95032, USA; 5 Endoscopy Center of Silicon Valley, Los Gatos, CA 95124, USA

**Keywords:** Crohn’s disease, Stem Cell Educator therapy, autoimmune diseases, IL-1β, CXCL16

## Abstract

Crohn’s disease is a chronic inflammation affecting the gastrointestinal tract. To date, patients are commonly treated with corticosteroids or more aggressive biologics for high-risk subjects. Stem Cell Educator therapy has been successfully utilized to treat patients with type 1 diabetes and other autoimmune conditions. A 78-year-old patient with long-standing Crohn’s disease received one treatment with the Stem Cell Educator therapy, followed by clinical, radiographic, pathological examinations and immune marker testing by flow cytometry. After the treatment with Stem Cell Educator therapy, the patient’s clinical symptoms were quickly improved with normal bowel movements, without abdominal pain or rectal bleeding. Flow cytometry analysis revealed a marked decline in inflammatory markers, such as the percentage of monocyte/macrophage-associated cytokine interleukin-1 beta (IL-1β)^+^ cells, which reduced from 94.98% at the baseline to 18.21%, and down-regulation of the percentage of chemokine CXCL16^+^ cells from 91.92% at baseline to 42.58% at 2-month follow-up. Pathologic examination of the biopsy specimens from colonoscopy five weeks and six months post-treatment showed ileal mucosa with no specific abnormality and no significant inflammation or villous atrophy; no granulomas were identified. A follow-up CT scan four and one-half months post-treatment showed no evidence of the previously seen stenosis of the ilio-colonic anastomosis with proximal dilatation. Stem Cell Educator therapy markedly reduced inflammation in the subject with Crohn’s disease, leading to durable clinical, immunological, radiographic, and pathological improvements.

## 1. Introduction

Crohn’s disease is one of the common inflammatory bowel diseases (IBDs) that may affect any part of the gastrointestinal tract, possibly displaying extraintestinal complications in some patients [1,2,3]. Similar to other autoimmune diseases, the potential causes of Crohn’s disease remain unclear and might be associated with genetic predispositions and environmental factors [4,5]. The prevalence and disease severity of Crohn’s disease are increasing post-COVID-19 pandemic [6,7,8]. To date, the clinical management options for Crohn’s disease are limited, commonly applying corticosteroids to treat flare-ups of symptoms (e.g., diarrhea, abdominal pain, and rectal bleeding) [1]. Additionally, the immune-suppressive biologics might be combined in severe subjects to block the inflammatory cytokines such as TNFα (e.g., Humira and Remicade) and the p40 subunit of receptors for IL-12 and IL-23 (e.g., Stelara) [1,3,9]. Therefore, the limits of conventional therapies highlight the need for novel approaches to treat Crohn’s disease.

Over the last 15 years, we have developed a novel technology designated the Stem Cell Educator (SCE) therapy through international multicenter clinical trials, which demonstrated the clinical safety and efficacy in multiple autoimmune diseases such as type 1 diabetes (T1D) [10] and alopecia areata (AA) [11]. With this technology, the patient’s immune cells are co-cultured with adherent cord-blood-derived multipotent stem cells (CB-SCs) in vitro. After overnight co-culture, the “educated” immune cells are re-infused into the patient’s circulation [12]. Recently, Stem Cell Educator therapy has been approved by the United States FDA for Regenerative Medicine Advanced Therapy (RMAT) designation. Here, we report the clinical safety and efficacy of Stem Cell Educator therapy to treat a patient with long-lasting established Crohn’s disease.

## 2. Results

### 2.1. Medical History and Diagnosis of Crohn’s Disease

A 78-year-old man had symptoms of Crohn’s disease since his late teens, with intermittent right lower quadrant abdominal pain and frequent loose stools. At age fifty-seven, he presented with severe anemia with hemoglobin level of 6.0 due to chronic blood loss. Barium enema was consistent with Crohn’s disease. The terminal ileum, cecum, and right colon were resected. Over the ensuing years, several strictures were surgically repaired.

In early 2024, symptoms of stricture recurred. Colonoscopy showed a severe, benign-appearing, intrinsic stenosis of the colo-ileo junction. Small erosions were noted at the junction. The colonoscope was unable to pass into the small intestine. The biopsy forceps were passed, and biopsies were conducted. Some inflammation was seen. There was contact mucosal bleeding. Some localized Crohn’s disease was seen. Contrast CT scan of the abdomen showed a loop of small bowel in the anterior pelvis that was distended to approximately 3.7 cm proximal to a short segment 1 cm region of narrowing and minimal bowel wall thickening. Similarly, there was a distended loop of small bowel in the right lower quadrant measuring up to 3.4 cm. Pathological examination of the biopsy specimens showed sections from the terminal ileum with superficial acute ileitis and increased plasma cells within the lamina propria. There was some mild villous blunting. The findings were compatible with idiopathic inflammatory bowel disease with minimal activity.

### 2.2. Clinical Safety and Improvements of Symptoms After Stem Cell Educator Therapy

On 15 October 2024, the subject received treatment with Stem Cell Educator therapy. The patient tolerated the procedure well without side effects and was discharged home on no medications other than vitamin supplements. The patient reported immediate improvements in bowel function, which continued to improve over several weeks, eventually returning to normal.

### 2.3. Markedly Reduced Monocyte/Macrophage-Associated Inflammatory Markers IL-1β and CD163 After Stem Cell Educator Therapy

**Figure 1:**
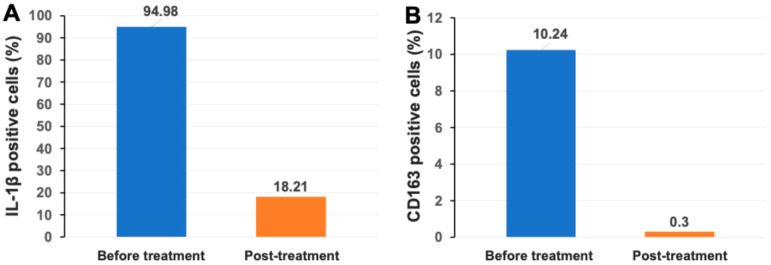
Decrease in monocyte/macrophage-associated inflammatory markers in PBMCs after the treatment with Stem Cell Educator therapy. (**A**) Marked decline in the percentage of IL-1β^+^ cells. (**B**) Reduced percentage of CD163^+^ cells.

IL-1β is one of the major cytokines normally released by monocytes and macrophages and contributes to the immune activation of T and B cells [13]. Flow cytometry analysis demonstrated that there was a high percentage of IL-1β^+^ cells in the patient’s PBMCs at 94.98% before the treatment. Notably, the percentage of IL-1β^+^ cells was markedly reduced to 18.21% two months after treatment with Stem Cell Educator therapy (Figure 1A). The activated monocyte/macrophage marker CD163 also declined from 10.24% at baseline to 0.3% post-treatment (Figure 1B). The data indicated the down-regulation of activated monocytes/macrophages after receiving Stem Cell Educator therapy.

### 2.4. Markedly Reduced Expression of Inflammatory Chemokine CXCL16 After Stem Cell Educator Therapy

**Figure 2:**
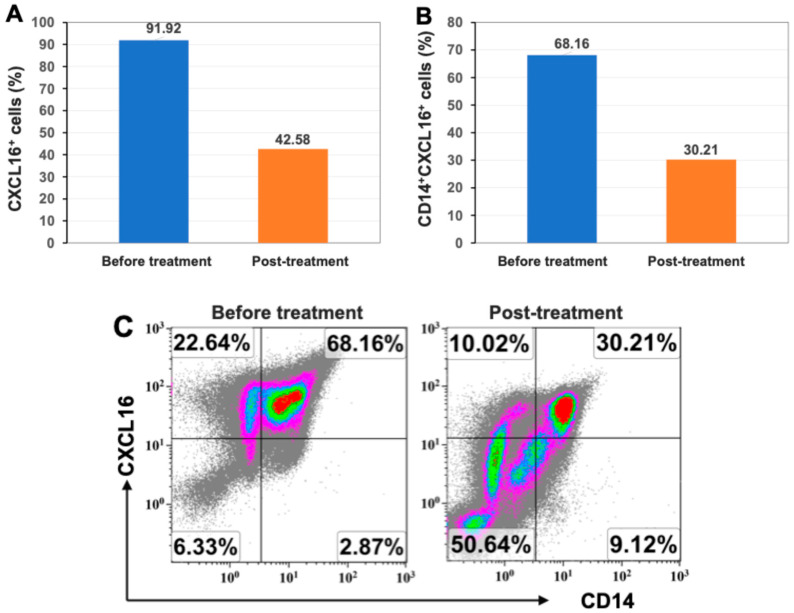
Reduced level of chemokine CXCL16 expression in PBMC after treatment with Stem Cell Educator therapy. (**A**) Percentage of total CXCL16^+^ cells was reduced. (**B**) Percentage of CD14^+^CXCL16^+^ monocytes was reduced. (**C**) Dot plot showed CD14^+^ monocytes reduced expression of CXCL16^+^. Different colors represent the densities and distributions of different cell populations. Isotype-matched IgGs served as negative controls for flow cytometry analysis.

The C-X-C motif ligand 16 (CXCL16) is a chemokine and is expressed by a variety of immune cells, such as monocytes/macrophages, dendritic cells, and B cells. Recent studies suggest the contribution of CXCL16 in the pathogenesis of autoimmune diseases such as Crohn’s disease, multiple sclerosis, rheumatoid arthritis, and lupus [14,15,16]. Flow cytometry showed that the patient’s PBMCs displayed high percentages of CXCL16^+^ cells, 91.92% at baseline. This was significantly reduced to 42.58% two months after treatment with Stem Cell Educator therapy (Figure 2A). CXCL16 is normally enriched in the monocytes/macrophages. Using the monocyte-specific marker CD14, further flow cytometry analysis demonstrated that the percentage of CD14+CXCL16+ monocytes was down-regulated from 68.16% at baseline to 30.21% at 2-month follow-up (Figure 2B,C). Additionally, the percentage of the costimulating molecule CD86 (B7-2) was reduced from 3.83% at baseline to 1.66% at 2-month follow-up. There were no marked changes in the costimulating molecules CD80 (B7-1, 0.00% vs. 0.01%), CD282 (TLR-2, 1.38% vs. 0.01%), and CD284 (TLR4, 0.01% vs. 0.01%) pre- and post-treatment. The data implied a decline in the infiltration and activation of monocytes/macrophages.

### 2.5. Up-Regulation of Percentage of Regulatory T Cells (Treg) After Stem Cell Educator Therapy

**Figure 3:**
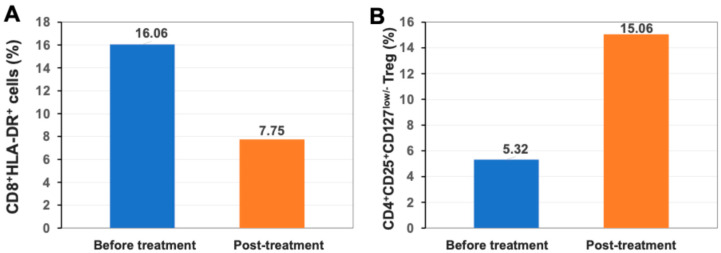
Modulation of T cells after treatment with Stem Cell Educator therapy. (**A**) Down-regulation of percentage of activated CD8^+^HLA-DR^+^ T cells. (**B**) Up-regulation of percentage of CD4^+^CD25^+^CD127^low/−^ Tregs.

Using HLA-DR as a marker for activated T cells, flow cytometry showed that the percentage of activated CD8^+^HLA-DR^+^ T cells was reduced from 16.06% at the baseline to 7.75% at 2-month follow-up (Figure 3A). There were no changes in the percentage of activated CD4^+^HLA-DR^+^ T cells after treatment (11.49% at baseline vs. 12.91% at 2-month follow-up). Additionally, flow cytometry demonstrated that the percentage of CD4^+^CD25^+^CD127^low/−^ Tregs was increased from 5.32% at the baseline to 15.06% at 2-month follow-up (Figure 3B), which might contribute to the down-regulation of activated CD8^+^ T cells.

### 2.6. Pathologic and Radiographic Improvements After Stem Cell Educator Therapy

**Figure 4:**
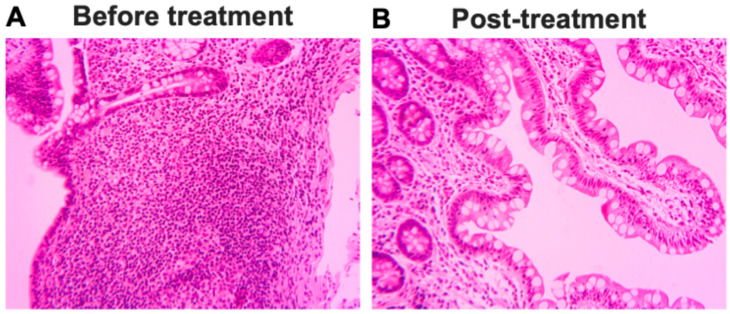
Pathological changes in terminal ileum biopsy after receiving Stem Cell Educator therapy for five weeks. (**A**) Representative image from one of four pieces of terminal ileum biopsies showed superficial acute ileitis and increased infiltrated immune cells within lamina propria before treatment. (**B**) Representative image from one of five pieces of terminal ileum tissues showed ileal mucosa with no specific abnormality after treatment with Stem Cell Educator therapy for 5 weeks. A few lymphocytes presented without significant inflammation or villous atrophy. Microscopy images were taken via inverted microscope with AmScope camera MU1000 using AmLite software v4.11. Magnification: ×200.

At a follow-up colonoscopy five weeks post-stem cell treatment, the stenosis at the ileo-colonic anastomosis was improved, and the colonoscope was able to pass through it by 1–2 cm. Pathologic examination of the biopsy specimens showed ileal mucosa with no specific abnormality and no significant inflammation or villous atrophy (Figure 4). No granulomas were identified, and no inflammation similar to that seen in the prior biopsy was seen in the 5-week post-treatment ileal biopsy (Figure 4B). A follow-up CT scan four and one-half months post-stem cell treatment showed no evidence of small bowel obstruction. No focal segmental small bowel wall thickening was identified. Previously noted loops of small bowel in the anterior abdomen measuring up to 3.7 cm were now within normal limits. A follow-up colonoscopy six months post-Stem Cell Educator therapy showed that the stenosis at the ileo-colonic anastomosis was further improved, and the colonoscope was able to be passed 10 cm into the terminal ileum. Pathologic examination of the biopsy specimens showed minimal non-specific chronic inflammation, providing no evidence of active idiopathic inflammatory bowel disease. These results show that Stem Cell Educator therapy for Crohn’s disease can provide long-term benefit up to at least six months.

## 3. Discussion

Crohn’s disease is a chronic inflammatory disease of the gastrointestinal tract. The current clinical study demonstrated a marked reduction in inflammation in a patient with long-standing Crohn’s disease, leading to clinical, immunological, radiographic, and histological improvements after one treatment with Stem Cell Educator therapy. Specifically, the activated monocyte/macrophage-associated markers IL-1β and CXCL16 were declined post-treatment, highlighting the potential of Stem Cell Educator therapy for the clinical management of patients with Crohn’s disease.

Monocytes/macrophages are antigen-presenting cells distributed in almost every organ. Intestinal macrophages play essential roles in maintaining gastrointestinal homeostasis, the balance of the gut immune system, and the regulation of gastrointestinal motility and secretion [17,18,19]. Clinical and animal studies have demonstrated the involvement of intestinal macrophages in the pathogenesis of Crohn’s disease and colitis [20]. Current clinical studies have revealed that there are high-profile inflammatory markers in subjects with Crohn’s disease, specifically activated monocyte/macrophage-associated markers such as IL-1β and chemokine CXCL16. After treatment with Stem Cell Educator therapy, these inflammatory markers were substantially reduced, as confirmed by flow cytometry analysis, leading to the clinical, pathological, and radiographic improvements. IL-1β, produced by monocytes/macrophages, is a master regulator of inflammation by controlling a variety of innate immune processes [21]. Interestingly, this subject displayed a high expression of IL-1β, similar to patients with T1D and other autoimmune- and inflammation-associated diseases. This suggests that the activated monocyte/macrophage system might be the common pathological feature in these autoimmune conditions. Our previous studies revealed that CB-SCs could release extracellular vesicles (EVs, exosomes) [22], which could target monocytes and change the differentiation of macrophages from M1 (inflammatory) to M2 (anti-inflammatory), contributing to a reset of the immune system.

Additional clinical and animal studies have indicated various types of immune cells involved in the pathogenesis of Crohn’s disease, such as T cells [23,24,25], B cells [26,27,28], and NKT cells [29,30,31]. Flow cytometry showed a high level of activated CD8^+^ T cells in this patient before treatment, which was markedly reduced after Stem Cell Educator therapy. Notably, Stem Cell Educator therapy displayed a range of immune modulations via multiple molecular and cellular mechanisms in different compartments of immune cells, such as inducing immune tolerance through the expression of autoimmune regulators (AIREs) in CB-SCs [10]; down-regulating activated T cells and B cells through cell–cell interactions mediated by PD-L1 (CD274) and HVEM (CD270), which are expressed on CB-SCs and whose ligands, PD-1 and BTLA, are expressed on immune cells (T cells, B cells, monocytes, DC, and granulocytes) [11]; suppressing the proliferation of activated B cells; and reducing antibody production through a galectin-9-mediated cell–cell contact mechanism [32]. Therefore, Stem Cell Educator therapy may hold great promise in treating patients with Crohn’s disease and ulcerative colitis and improving their quality of life.

## 4. Materials and Methods

### 4.1. Patient and Treatment with Stem Cell Educator Therapy

A 78-year-old man with Crohn’s disease was enrolled and received treatment with Stem Cell Educator therapy via the Right-to-Try Act program at Throne’s Outpatient facility (Paramus, NJ, USA) according to the US FDA-approved clinical protocol for type 1 diabetes (IND 19247, ClinicalTrials.gov Identifier: NCT04011020). The consent form and clinical protocol were approved by the central Institutional Review Board (IRB) at Advarra (Columbia, MD, USA) on 24 May 2024, with the approval code MOD02100578. The signed written informed consent form was obtained from the subject. Throne has received Blood Bank License approval for the Stem Cell Educator therapy from the Department of Health in New Jersey. The objective of this study was to evaluate the feasibility and clinical efficacy of Stem Cell Educator therapy for the treatment of Crohn’s disease.

The patient received the Stem Cell Educator therapy as previously described [12]. In brief, when the confluence of CB-SC cultures was more than 80%, they were utilized to treat the patient’s peripheral blood mononuclear cells (PBMCs) isolated by using the blood cell separator Spectra Optia Apheresis System (Terumo BCT, Lakewood, CO, USA) at Throne’s Outpatient facility. The leukapheresis product was transported at ambient temperature and processed at Throne’s Good Manufacturing Practices (GMP) facility (Paramus, NJ, USA). After overnight co-culture, the Stem Cell Educator-treated PBMCs were collected into a 1000 mL empty IV bag (ICU Medical, San Clemente, CA, USA). The volume of the final product was about 750 mL/bag. After being shaken and mixed, 25 mL volume of cell suspension was sampled from the bag of final product for quality control (QC) testing, such as cell viability, concentration, endotoxin level, and potency markers, by flow cytometry, and then released for infusion at Throne’s outpatient facility according to the FDA-approved protocol. The patient was scheduled to perform immune cell marker follow-up studies on the 1st, 3rd, and 6th month after treatment with Stem Cell Educator therapy.

### 4.2. Flow Cytometry

Patient’s PBMCs were utilized to examine the changes in immune markers by flow cytometry pre- and post-treatment with Stem Cell Educator therapy. PBMCs were isolated with Ficoll-hypaque (γ = 1.077, GE Health), and red blood cells were lysed using the ammonium–chloride–potassium (ACK) lysis buffer (Lonza, Walkersville, MD, USA). Flow cytometry analysis was performed as previously described [12] using the following antibodies: human regulatory T cell cocktail (BD Biosciences, Franklin Lakes, NJ, USA), pacific blue (PB)-conjugated mouse anti-human CD3, Spark Violet 538 anti-human CD4, APC/Fire 750 anti-human CD8, Spark Violet™ 538 anti-human CD14, PerCP/Cyanine5.5 anti-human CD80, PB-conjugated mouse anti-human CD86, PerCP/Cyanine5.5-conjugated mouse anti-human CD163 FITC-conjugated mouse anti-human HLA-DR mAb, PB-conjugated mouse anti-human IL-1β mAb, PE-conjugated mouse anti-human CXCL16, PE-conjugated anti-human CD282 (TLR2), and APC-conjugated anti-human CD284 (TLR4) mAbs were purchased from (Biolegend, San Diego, CA, USA). Isotype-matched immunoglobulins (IgGs) served as controls. For intra-cellular staining, cells were fixed and permeabilized according to the PerFix-nc kit (Beckman Coulter, Brea, CA, USA) according to the manufacturer’s recommended protocol. After staining, cells were collected and analyzed using a Gallios Flow Cytometer (Beckman Coulter, Brea, CA, USA) equipped with three lasers (488 nm blue, 638 red, and 405 violet lasers) for the concurrent reading of up to 10 colors. The final data were analyzed using the Kaluza Flow Cytometry Analysis Software (Kaluza Analysis 2.1, Beckman Coulter).

## Data Availability

The data that support the findings of this study are available from the corresponding author upon request.

## Abbreviations

CB-SCs, cord blood-derived stem cells; CXCL16, C-X-C motif ligand 16; FDA, Food and Drug Administration; IL-1β, interleukin-1β; IRB, Institutional Review Board; PBMCs, peripheral blood mononuclear cells; SCE, Stem Cell Educator; T1D, type 1 diabetes.
